# A public health approach to reducing violence within the CARICOM region

**DOI:** 10.3389/fpubh.2024.1344387

**Published:** 2024-02-15

**Authors:** Sandeep Maharaj, Randy Seepersad, Joanna Sooknanan, Simon Anderson, Darleen Franco, Amrica Ramdass, Terence Seemungal

**Affiliations:** ^1^Faculty of Medical Sciences, The University of the West Indies, St. Augustine, Trinidad and Tobago; ^2^Planetary Health Alliance, Boston, MA, United States; ^3^Criminology Unit, University of the West Indies, St. Augustine, Trinidad and Tobago; ^4^The University of the West Indies Open Campus, Kingston, Jamaica; ^5^Population Health Sciences, Faculty of Medical Sciences, University of the West Indies, Cave Hill Campus, Bridgetown, Barbados; ^6^The George Alleyne Chronic Disease Research Centre, A Unit of the Caribbean Institute for Health Research, Cave Hill, Bridgetown, Barbados; ^7^Primary Care and Public Health, Northwest Regional Health Authority, Port of Spain, Trinidad and Tobago; ^8^Faculty of Medical Sciences, St. Augustine, Trinidad and Tobago; ^9^Department of Clinical Medical Sciences, Faculty of Medical Sciences, University of the West Indies, St. Augustine, Trinidad and Tobago

**Keywords:** Caribbean, crime, homicide, public health, CARICOM

## Abstract

Widespread crime has become a worldwide problem so much so that violence is now ranked fourth globally in its contribution to disability-adjusted life years in the 10 to 24 age group. Homicides, a surrogate marker of violent crime, have shown an upward trend in almost all of the CARICOM countries, and homicide rates over the past 3 years have consistently increased, though the pattern of violence varies by country. This background has informed the need for greater emphasis on the need for a different approach to dealing with crime in the CARICOM region. The CARICOM governments recently hosted a symposium on crime and violence as a public health issue. The public health approach to crime has been used with measurable success in different parts of the world and, more recently in Trinidad, one of the CARICOM countries. The paper outlines the outcomes of the symposium and discusses its implications for the region.

## Introduction: violence in the CARICOM region

In the last few decades, the nature of crime worldwide has changed as crime has become more sophisticated, organized, and transnational. Crime has achieved macro-economic proportions while transforming “into a global business operating in collusion with legitimate activity” and, the CARICOM region has evolved from localized violence into a “widespread threat to the security of cities, states, even entire regions” (1). The 2019 Global Burden of Disease study has shown that violence in the 10 to 24 age group ranks fourth globally in its contribution to disability-adjusted life years (DALYs) (2).

Within the Caribbean – made up of mostly member states of the Caribbean Community (CARICOM) – criminal activities now include maritime piracy, arms, drug and human trafficking, money laundering, cybercrime, identity theft, extortion, and corruption. With some of the highest regional violent crime rates in the world (3) the Caribbean is one of the most dangerous parts of the Americas (4). A consideration of homicide rates (Table 1) across the 15 CARICOM member states shows Trinidad and Tobago, St. Lucia, St. Vincent and the Grenadines, and Jamaica having the highest homicide rates in the region. With homicide rates corresponding to the prevalence of violent crime in a country (6), Table 1 shows an increasing trend in violence across CARICOM.

**Table 1:** Number of homicides per 100,000 in CARICOM member countries, from 2019–2022 (United Nations population estimates were used to calculate 2022 rates) (5).

Countries	2019	2020	2021	2022
Antigua and Barbuda	3.1	9.2	16.0	17.2
Bahamas	24.2	18.6	29.2	32.0
Barbados	16.7	14.3	11.4	15.3
Belize	33.7	25.7	29.0	25.0
Dominica	19.4	19.4	13.8	23.3
Grenada	14.2	14.2	4.0	N/a
Guyana	17.3	20.0	15.2	15.1
Haiti	9.5	12.1	13.7	18.8
Jamaica	45.2	44.7	49.4	52.9
Montserrat	0.0	0.0	N/A	N/A
St. Kitts and Nevis	22.6	18.8	29.0	23.1
St. Lucia	28.9	28.3	39.0	42.3
St. Vincent and the Grenadines	17.1	26.1	30.7	40.3
Suriname	5.1	9.4	5.7	7.7
Trinidad and Tobago	38.4	28.4	32	39.4

Although the CARICOM member states are experiencing an increase in violent crime, the types of violence vary by country. For example, most homicides in Trinidad and Jamaica are gang-related (7) and there is a well-established local link between violent crime, gangs, guns and the illegal drug trade (8). Besides the trafficking of narcotics, gangs are also thought to be associated with other crimes that accompany the drug trade, such as kidnapping, robberies, firearms activity, and the violence associated with turf wars with rival gangs (8). With increasingly sophisticated and high-calibre firearms and ammunition being smuggled into countries, the rate of violent deaths in CARICOM member states is almost three times the global average with firearms involved in more than half of all homicides in the region (9). While gang violence has been highlighted as one of the most important factors responsible for violence in Jamaica and Trinidad and Tobago, it is of increasing concern in Barbados and other CARICOM nations.

Arising from this alarming increase in violence, CARICOM recently hosted a symposium on crime and violence as a public health issue. This symposium was attended by the heads of government of CARICOM member states, as well as technocrats, the police service, prison service, NGOs, and other persons and organizations relevant to crime and security from across the Caribbean region. The symposium was organized and hosted by CARICOM and held in Trinidad and Tobago on April 17–18, 2023. This article gives an overview of this approach and the recommendations from the symposium while considering the unique challenges faced by CARICOM member states. While violent crime is a subset of violence since not all crimes (for example, white collar crime) are associated with violence, the crimes considered in this article are violent crimes.

## The public health approach and recommendations from the symposium

The United Nations considers any homicide rate of 10 per 100,000 citizens or above to be an “epidemic.” The data in Table 1 show that 13 out of the 15 CARICOM member states have consistently high murder rates according to this criterion. A consistently high murder rate, as well as well as the consistent increase in violent crimes among CARICOM member countries, suggest that the approaches which have been taken to deal with crime in the Caribbean have not been successful. Countries across the region have placed emphasis on suppressive approaches, and rely on the actions of the police, courts and prisons to deal with the crime situation.

The failure of suppressive approaches is not surprising as such approaches do not address the root causes of crime and violence. While there is a wealth of Caribbean research on root causes, with very few and isolated exceptions, these have not been used in the development of interventions, and have not been focused upon when attempting to deal with crime in the region. Unfortunately, leaving the root causes intact means that the conditions which drive crime and violence will persist, and so too will crime and violence. Root causes in the region may include low educational attainment, poverty, inequality, problems in the family, a lack of welfare services, and the like. The root causes have a history which is situated in the colonial past of the region where the people and resources of the region were utilized to drive the industrial age in Europe and the UK. Andre Gunder Frank’s dependency theory represents an eloquent statement which outlines the processes which led to the underdevelopment of the Caribbean region, and which resulted in a lack of resources to transform the countries of the region into developed nations.

A public health approach is one which is applicable to addressing root causes. “The principles of public health provide a useful framework for both continuing to investigate and understanding the causes and consequences of violence and for preventing violence from occurring through primary and secondary prevention programs, policy interventions, and advocacy” (10). This approach emphasizes prevention and intervention. The public health approach can help reduce crime by treating crime and violence like a disease and looking for innovative ways to prevent this “disease” from spreading. In fact, several mathematical models of crime as a disease already exist and they allow for the examination of preventive approaches in different scenarios (11). Such analytic modelling based on data collected should assist in the design of public health approaches to violence reduction.

The public health approach to solving problems consists of four basic elements (12).

1. Define and monitor the problem: Understanding the who, what, when, where, and how of violence is the first step in stopping it. Data from police reports, medical examiner files, vital records, hospital charts, registries, population-based surveys, and other sources are analyzed.
2. Identify risk and protective factors: Understanding what variables protect persons from or put people at risk of experiencing or perpetrating violence is also critical. Risk and protective factors aid in determining where prevention efforts should be directed.
3. Develop and test prevention strategies: For designing preventative initiatives, research data and findings from needs assessments, community surveys, stakeholder interviews, and focus groups are useful. After programs are implemented, they are rigorously evaluated to determine their effectiveness.
4. Implementation: Once prevention programs have been proven to be effective, they must be implemented and widely adopted. Techniques for promoting wider adoption include training, networking, technical assistance, and evaluation.

One of the themes arising from the 2023 CARICOM Heads of Government symposium was the declaration to treat crime and violence as a public health challenge. This contrasted with an earlier approach in 2013, in which there was a marked emphasis on a criminal justice approach with recommendations that spanned from financial penalties to intelligence collecting and public awareness (13). However, perhaps due to ineffective implementation, this approach appeared to make little difference to crime across the region. Despite this, it is not being said here that a public health approach should be used as a replacement to the more traditional law enforcement-based approaches. Indeed, both approaches complement each other and should be used in tandem with each other.

Out of the 15 declarations from the CARICOM symposium, the following four aligned with the public health approach:

**Empower and engage** young people as positive content developers to offset the negative impact of social media and engage with the creative industries to re-engineer culturally acceptable norms;
**Promote** public awareness and education campaigns in our communities, that challenge harmful beliefs, attitudes, and behaviors that contribute to crime and violence;
**Work** with all sectors and institutions to improve equitable access to services and options for rehabilitation and reintegration into society, psychosocial support and parental education, addressing domestic violence, and integrating mental issues to treat crime and violence;
**Develop and implement** targeted programs and strategies to address young vulnerable youth at risk of becoming perpetrators and victims of crime.

## The public health approach to crime in CARICOM

While the region has generally adopted a punitive approach at the expense of preventative approaches, the public health approach has already been implemented in CARICOM countries. For example, an adaptation of the Cure Violence program, which utilizes a community-based, public health approach to prevent the “transmission” of violent crime and the reduction of violence (14), was implemented in Trinidad from July 2015 to August 2017. An analysis of its effectiveness determined an overall reduction in violent crime by approximately 38% and a decrease in shooting injuries by approximately 39% (15). This approach was recently relaunched in Trinidad and Tobago and was rebranded as Project Building Blocks. More recently, in 2022, the public health approach was used in the Youth Resilience, Inclusion and Empowerment (Y-RIE) project. This is a 5-year USAID-funded project which aims to target at-risk youth in Grenada, Guyana and St. Lucia. Through the use of a custom-developed risk assessment instrument, the project aims to target at-risk youth in high crime communities in each country, and to provide interventions and services which are based on an empirical assessment of risk and protective factors in each of the countries.

Still, much needs to be done in the Caribbean region to ensure deeper understanding, acceptance and usage of the public health approach in managing the crime situation. Governments of the region have taken a progressively punitive approach to dealing with crime. Crime is a highly politicized issue in the region, and as such, governments of the region do not want to appear to be “soft on crime.” Public opinion in the Caribbean, unfortunately, has been driven by news media portrayals of crime which typically recommend punitive approaches. In their bid to ensure that they stay in power, Caribbean governments have consistently relied on approaches which increase fines and sentences, and which result in a widening net which extends the range of things which are criminalized. Given the above, much still needs to be done to develop the commitment by Caribbean citizens and governments to the public health approach. Until this is done, punitive approaches will still take precedence.

## Discussion

Violence affects not only its victims but may result in a decline in tourism, increased emigration with a resulting brain drain, a reluctance to invest locally by foreign investors, and a general feeling of disquiet and fear by the population. Apart from the direct effect on victims, exposure to crime and violence can lead to an increased risk of asthma, hypertension, cancer, stroke, depression, and post-traumatic stress disorder, as well as a range of negative mental health outcomes (16, 17). These consequences in turn increase the economic burden not only on the health care systems but also on the national economy in terms of absenteeism, loss of productivity, expenses within the criminal justice system, and other costs.

Despite being overburdened, overwhelmed, and under-resourced, there is still an overdependence and overconfidence in the criminal justice system as a panacea for dealing with criminal behavior. This may result in scant consideration of other approaches that may be more effective in reducing certain types of criminal behavior, like youth violence and violence against women, in a cost-effective, timely manner (6). The core problem with an over-reliance on suppressive approaches to crime prevention is that they leave the root causes or drivers of the problem intact and only come into play after offenses have been committed. Once the drivers of the problem continue to exist, the problem will persist. Despite this, as stated above, there are some examples of preventative approaches that have been used in the Caribbean. Unfortunately, suppressive Also, dealing with the preventative factors of crime, the region continues to be burdened with the issues of poverty and social inequity some of which stem from the historic colonial arrangement which the current Caribbean region was founded upon. The need for reparatory justice is clear and removing the burden of debt placed on them (18). In combination with the above, welfare policies that prioritize housing, education and affordable medical services will help mitigate crime. Cuba has one of the lowest crime rates in Latin America and the Caribbean and they use a social community based approach which needs to be further investigate (19). approaches continue to be far more popular and receive the bulk of funding.

Also, dealing with the preventative factors of crime, the region continues to be burdened with the issues of poverty and social inequity some of which stem from the historic colonial arrangement which the current Caribbean region was founded upon. The need for reparatory justice is clear and removing the burden of debt placed on them (18). In combination with the above, welfare policies that prioritize housing, education and affordable medical services will help mitigate crime. Cuba has one of the lowest crime rates in Latin America and the Caribbean and they use a social community based approach which needs to be further investigate (19).

The Caribbean has a long history of addressing and mitigating key public health challenges through effective, evidence-based intervention programs. The region has had to deal with COVID-19 as a region, not as individual nations, and was successful. The success in the medical arena suggests that the Caribbean can apply the same approach to combating violence from a public health standpoint. In a similar manner to the response to COVID-19, by reducing the numbers susceptible to becoming infected and the numbers infected and going into hospital, then the “infection” may be controlled.

An important feature of the public health approach is that public health is always viewed from the perspective of the determinants of health. Traditional health conditions are impacted by several things. These factors include poverty, access to social services, and marginalization. These are also factors that can affect crime and violence. Because behavior is at the heart of health, this perspective should consider what drives people to behave in ways that allow crime and violence to thrive. Resources must be allocated to collecting and analyzing data in order to obtain a better understanding of the social and economic causes of violence and to develop empirically based interventions. It is, however, important to stress the need for research in determining which risk and protective factors may vary geographically, and with the type of crime that is prevalent in any one country.

Interventions based on empirical data will remove the guesswork about which factors should be focused upon in designing preventative and pre-emptive intervention measures aimed at reducing and preventing the spread of violence. This can involve a coordinated effort to examine regional data, as well as conduct research to determine what types of interventions are likely to be successful in a Caribbean context, and then ensuring resource allocation so that promising interventions can be piloted, and later expanded if they prove to be effective. This also speaks to the issue of monitoring and evaluation, which is a critical element for all crime reduction initiatives.

We posit that violence has become the norm in many Caribbean societies and addressing violence and criminality is now a major public health issue. Because it affects all of us, the response must be one that immunizes people, at-risk communities, and at-risk individuals against the tendency to adopt violence as a way of life. CARICOM has demonstrated a history of success where health is concerned and is well-positioned to take the lead in a regional effort to combat the scourge of crime and violence that affects so many nations in the Caribbean region. The lessons learned from the health sector can be successfully applied in the fight against crime.

## Data availability statement

Publicly available datasets were analyzed in this study. This data can be found here: https://www.statista.com/statistics/947781/homicide-rates-latin-america-caribbean-country/.

## Author contributions

SM: Writing – original draft. RS: Writing – original draft. JS: Writing – original draft. SA: Writing – review & editing. DF: Writing – review & editing. AR: Writing – original draft. TS: Writing – original draft.
